# Bioactivity and mechanisms of flavonoids in decreasing insulin resistance

**DOI:** 10.1080/14756366.2023.2199168

**Published:** 2023-04-10

**Authors:** Min Zhou, William H. Konigsberg, Canhua Hao, Yinbo Pan, Jie Sun, Xiaojing Wang

**Affiliations:** a School of Parmacy and Pharmaceutical Sciences & Institute of Materia Medica, Shandong First Medical University & Shandong Academy of Medical Sciences, NHC Key Laboratory of Biotechnology Drugs (Shandong Academy of Medical Sciences), Key Lab for Rare & Uncommon Diseases of Shandong Province, Ji’nan, Shandong, China; b Department of Molecular Biophysics and Biochemistry, School of Medicine, Yale University, New Haven, Connecticut, USA

**Keywords:** Flavonoids, insulin resistance, inflammation, obesity, diabetes

## Abstract

Flavonoids are ubiquitous compounds in nature and are found in many Chinese herbal medicines. Due to their biological activity, flavonoids show potential for decreasing insulin resistance (IR), thereby delaying the progression of diabetes and accompanying metabolic syndromes. This review focuses on the mechanisms of flavonoids decreasing IR: (1) the interaction between flavonoids and target proteins of the insulin signalling pathway; (2) bioactivities of flavonoids, such as anti-inflammatory, lipid-lowering and antioxidant. Meanwhile, we summarise the structural characteristics, structure activity relationships and biological activity of flavonoids, providing evidence for their potential in the treatment of IR. Here, we also analyse the potential and limitations of their therapeutic use.

## Introduction

Insulin resistance (IR) is a pathophysiological condition that refers to decreased sensitivity and responsiveness to insulin by its target organs. Insulin is a hormone secreted by pancreatic β-cells that regulates blood glucose levels, and its receptors are distributed throughout the body. However, the main targets are liver, fat, and skeletal muscle cells. The pathogenesis of IR includes abnormal secretion of pancreatic β-cells, changes in insulin receptor-related genes, and abnormalities in downstream signalling pathways^1^. This chronic metabolic disorder often appears in the early stage of the disease and, if not detected or treated, can lead to type 2 diabetes and metabolic syndromes. Several studies have found that IR may be present several years before the diagnosis of type 2 diabetes^2^. Epidemiological studies have shown that IR can pose a significant threat to health. It could lead to dyslipidemia, obesity, hypertension, diabetes, and cardio and cerebrovascular diseases^1^. Adipose tissue IR plays a vital role in obesity-related IR^2^. Cytokines and adipokines released by adipose tissue cause the body to develop chronic low-grade inflammation and IR^1^^,^^3^. In addition, many critical pathways in lipid metabolism become essential targets for decreasing IR^2^. IR disrupts glucose uptake in muscle while promoting gluconeogenesis in the liver. Increased metabolic demand induces the development of inflammation and stress-mediated IR, inhibiting insulin action in target organs or tissues^4^. There is an essential link between obesity, inflammation and the development of IR^5^^,^^6^. A wide variety of drugs are currently approved for the treatment of diabetes, but the response and efficacy are patient dependent. It has become an essential issue in medicine and related fields to effectively decrease IR and delay the development of diabetes and its complications^2^^,^^7^. In order to achieve this goal, many natural drugs, and their derivatives have been studied, among which flavonoids rich in biological activity can provide significant help.

**Figure 1.**
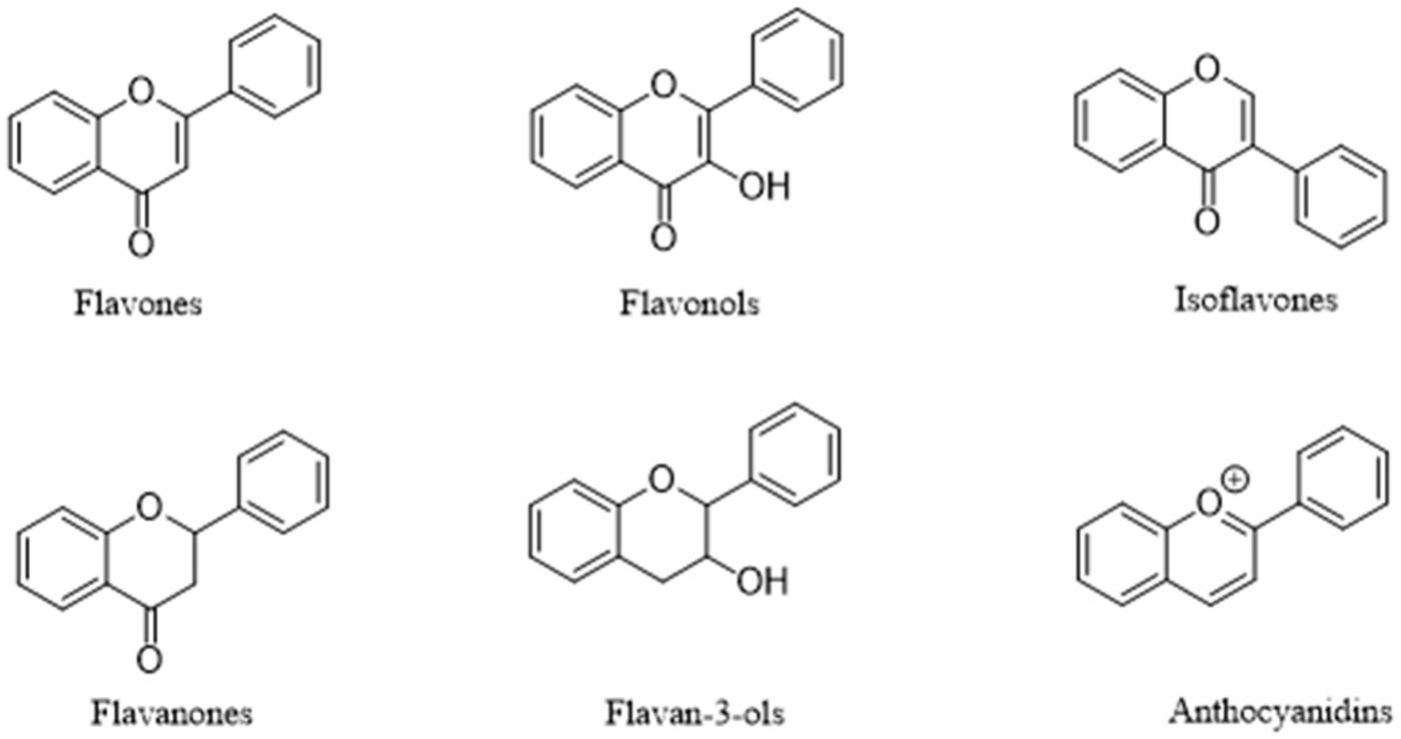
Chemical structures of different subclasses of flavonoids.

Flavonoids, are a class of natural products that have proven medicinal value. At the same time, flavonoids are able to maintain health and alleviate pre-disease states from a dietary perspective. Chemically, flavonoids consist of a basic three-ring nucleus, and the basic structure encompasses two benzene rings linked through a heterocyclic pyran or pyrone (with a double bond) ring in the centre. They are categorised into a number of subclasses according to their substitutions, and these are flavones, flavanols, flavonols, flavanones, isoflavones, and anthocyanidins (Figure 1). Flavonoids extracted from various plants have a wide range of biological activities, such as antioxidant, anti-inflammatory, anti-viral, anti-atherosclerotic, anti-diabetic and antitumor, and effects thus have great potential benefits for health^5–9^. In *in vitro* and in animal studies, numerous reports also support the beneficial effects of flavonoids in decreasing IR and diabetes^10–12^. In this review, we will systematically deal with the mechanisms by which flavonoids decrease IR, and summarise the biological activities of various flavonoids as well as their clinical applications and medicinal prospects in alleviating IR.

## Molecular mechanisms involved in insulin resistance

**Figure 2.**
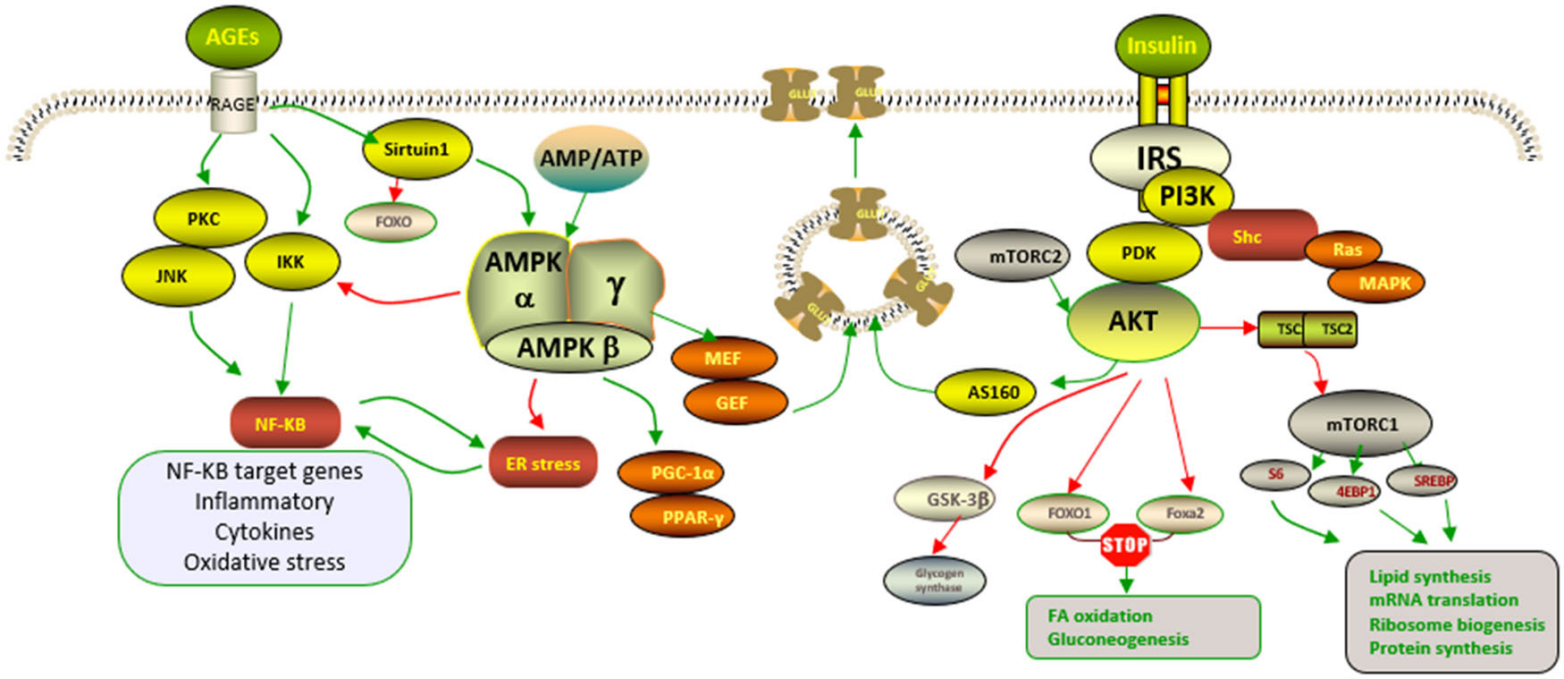
Various pathways involved in dysregulation of insulin signalling^3^. Activation of ir by their ligands initiates a cascade of phosphorylation events. And irs1 activates the PI3K-Akt pathway by recruiting and activating PI3K, which phosphorylates the serine/threonine residue of protein kinase B (Akt). Akt regulates the translocation of GLUT4 to the cell surface through AS160. Binding of AGE to its receptor RAGE impairs insulin signal by triggering a range of signalling pathways, including JNK, NF-κB, and activation of PKC. Red indicates inhibitory effects and green indicates positive effects.

**Figure 3.**
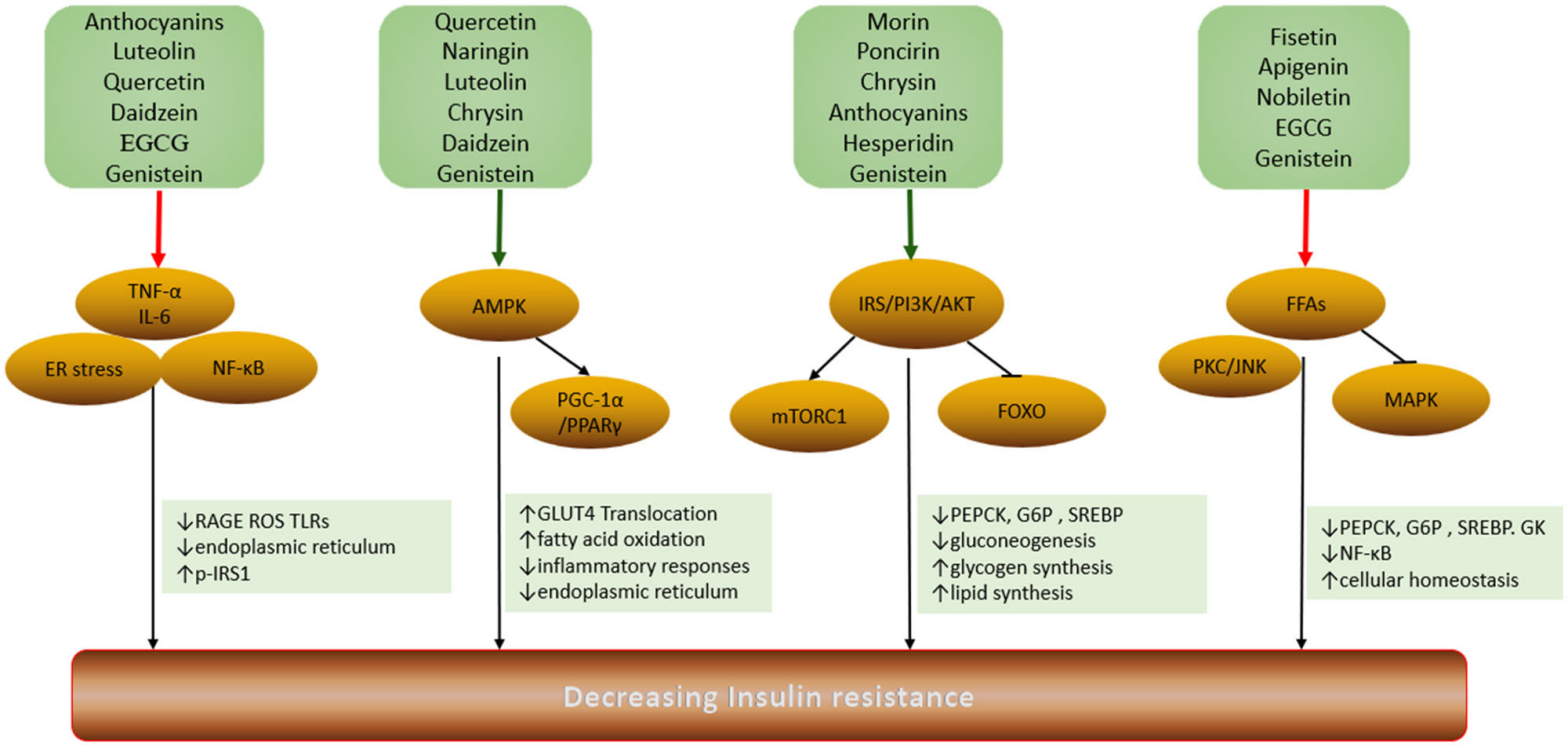
Sites of flavonoid action on insulin resistance (IR). Flavonoids induce insulin receptor and IRS phosphorylation and activate PI3K/Akt pathway and AMPK, promoting GLUT4 translocation. The PI3K/Akt pathway activated by flavonoids decreases PEPCK and G6P expression, suppressing gluconeogenesis and promoting glycogen synthesis. Flavonoids reduce the levels of FFAs and inflammatory factors, reducing the negative effect on insulin signal transduction by JNK, NF-κB and PKC. Red indicates inhibitory effects and green indicates positive effects.

IR is the result of a complex combination of metabolic disorders, lipotoxicity, glucotoxicity and inflammation. The ultimate embodiment of IR lies in altered insulin signal transduction mechanisms (Figure 2). Insulin plays a role in target organs or target tissues through signal transduction, particularly liver, skeletal muscle and adipose tissue, which play unique roles in regulating human metabolism^3^. IR occurs when the body does not respond adequately to normal levels of insulin. IR involves multiple molecular and pathophysiological mechanisms, the cause of which may be formed through both innate and acquired factors^4^. Common genetic factors include mutations and polymorphisms in insulin receptors, glucose transporters and signalling proteins involved in insulin signalling. Acquired causes of IR include obesity, lack of exercise, chronic inflammation, advanced glycation end products (AGEs), excess free fatty acids (FFAs), psychological stress, smoking, alcohol consumption or certain drugs^3^. Flavonoids alleviate IR by acting on target proteins in the insulin signalling pathway. The biological activities of flavonoids such as anti-inflammatory, lipid-lowering and oxidative stress relief play a vital role in decreasing IR and managing metabolic syndrome (Figure 3).

### PI3K/Akt signaling pathway

The phosphatidylinositol 3-kinase (PI3K)/Akt pathway is a classical insulin signal transduction pathway. First, insulin binds to the α subunit of insulin receptor, activating the tyrosine-protein kinase of its β subunit. In turn, insulin receptor substrates (IRS) are activated by activated tyrosine-protein kinases. Shc protein activates the Ras-mitogen-activated protein kinase (MAPK) pathway, whereas IRS proteins activate the PI3K-Akt pathway mainly by recruiting and activating PI3K. The PI3K-Akt signalling pathway mediates most of the metabolic actions of insulin, regulating glucose transport, biosynthesis, gluconeogenesis, and glycogen synthesis; it also plays a vital role in the control of the cell cycle and survival^3^.

PI3K is a heterodimer with serine kinase activity capable of inducing the production of a second messenger phosphatidylinositol trisphosphate (PIP3), which interacts with other signalling proteins. As an important effector molecule, PI3K is a key regulatory node in converting extracellular signals activated by insulin and/or growth factors into intracellular activities^13^. The serine/threonine protein kinase Akt (Protein Kinase B, PKB) is composed of three isoforms, Akt1, Akt2, and Akt3. Among all isoforms of Akt, Akt2 is the most important isoform in insulin-mediated glucose uptake and lipid metabolism^4^. Phosphoinositide-dependent protein kinase-1 (PDK1) activates Akt by phosphorylating the threonine residue of Akt; subsequently, mammalian target of rapamycin complex 2 (mTORC2) completes the activation process of Akt by phosphorylating the serine residue of Akt^3^. Akt inhibits lipid breakdown by activating mTORC1 and promotes lipid synthesis by regulating sterol regulatory element-binding protein (SREBP) substrates. Activation of S6K1 by mTORC1 not only increases mRNA production and translation, but also participates in negative feedback regulation of insulin signalling through serine/threonine phosphorylation of IRS^14^. Targeting mTOR can disable the negative feedback loop, thereby reversing IR and helping to prevent and/or treat diabetes.

Akt interacts and phosphorylates the forkhead box transcription factor (FoxO) proteins that are involved in regulating the expression of lipogenic and gluconeogenic genes, especially FoxO1 and FoxO3. Akt attenuates FoxO1 transcriptional activity by inhibiting its translocation to the nucleus, thereby reducing gluconeogenesis^3^^,^^15^. Gluconeogenesis is essential for glucose homeostasis and renal gluconeogenesis is one of the major pathways of endogenous glucose production. The liver, kidney, and intestine are organs that express the key gluconeogenic enzymes, including phosphoenolpyruvate carboxykinase (PEPCK), fructose-1,6-bisphosphatase (FBPase), and glucose-6-phosphatase (G6Pase)^16^. PEPCK expression is markedly increased compared with FBPase and G6Pase levels under insulin-resistant conditions^17^. PEPCK and G6Pase have been shown to be transcriptionally regulated by a complex network of transcription factors and co-factors, including CREB (cAMP-response element binding protein), hepatocyte nuclear factor-4α and FOXO1^16^^,^^18^.

Akt directly phosphorylates AS160, limiting the activity of Rab-GTPase activating protein, leading to glucose transporter 4 (GLUT4) translocation and glucose uptake. GLUT4 is the major insulin-responsive glucose transporter and is found in adipose tissue, skeletal muscle, and cardiac muscle. Insulin deficiency or resistance can lead to loss of GLUT4 expression in skeletal muscle due to transcriptional repression. GLUT4 enhancer factor (GEF) and myocyte enhancer factor 2 (MEF2) dynamically control GLUT4 gene expression^19^. GLUT2 plays a critical role in the reabsorption of glucose and is mainly found in hepatocyte membranes, pancreatic β-cells^20^.

Any defect in the PI3K/Akt signalling pathway ultimately decreases glucose uptake in insulin-sensitive tissues, leading to IR. As such, targeting regulatory proteins upstream and downstream of the insulin signalling pathway can decrease IR.

### AMPK pathway

AMP-activated protein kinase (AMPK) is a ubiquitously expressed serine/threonine kinase with a heterogeneous trimeric structure consisting of one catalytic subunit (α) and two regulatory subunits (β and γ). As a metabolic regulator, AMPK is normally activated at lower energy states, thereby catalysing the generation of ATP^3^^,^^4^. In response to metabolic stress, AMPK is turned on to activate many downstream targets, mediating dramatic changes in cellular metabolism, cell growth and other functions^13^. Cellular energy sensors sirtuin1 and AMPK regulate each other and are associated with a wide range of beneficial effects in maintaining glucose homeostasis in insulin-resistant states. AMPK activates PGC-1α (peroxisome proliferator-activated receptor gamma, PPAR-γ, coactivator 1α), a master regulator of mitochondrial biogenesis. Also, AMPK activates PGC-1α via sirtuin1^21^. PGC-1α is a transcription coactivator that interacts with a wide range of transcription factors and participates in a variety of biological reactions^22^. Transcriptional coactivators refer to proteins or protein complexes that increase the probability of gene transcription by interacting with transcription factors, but do not themselves bind to DNA in a sequence-specific manner. PPARs (PPAR-α, PPAR-δ and PPAR-γ) are members of a relatively large family of nuclear receptors, all of which are transcriptionally coactivated by PGC-1α. PPAR-γ is essential for adipogenesis and differentiation, whereas PPAR-α and PPAR-δ play vital roles in fatty acid oxidation control^22^^,^^23^. In addition, AMPK can activate the transcription of GEF and MEF2 through phosphorylation, up-regulating GLUT4 expression^15^.

AMPK can also play a vital role in glucose uptake by phosphorylating previous metabolic substrates and transcriptional regulators, promoting mitochondrial biogenesis and fatty acid oxidation, while inhibiting the synthesis of fatty acids, cholesterol and proteins. AMPK downregulates the expression of lipogenic genes by directly phosphorylating lipogenic transcription regulators, Ser372 of SREBP 1c, and Ser568 of carbohydrate response element binding protein^24^. AMPK phosphorylates Ser79 of Acetyl-CoA Carboxylase1 (ACC1) and Ser221 of ACC2 to inhibit ACC by blocking dimerisation, thereby reducing ACC activity^25^. Activation of AMPK may improve insulin sensitivity by inhibiting *de novo* adipogenesis and increasing fatty acid oxidation, as well as by reprogramming liver cells to reduce lipid production. Activated AMPK in adipose tissue may improve insulin sensitivity by supporting mitochondrial integrity and preventing fatty acid redirection to liver and/or skeletal muscle by maintaining adipose tissue re-esterification and brown adipose tissue function^24^^,^^25^. In addition, AMPK also suppresses inflammatory responses, counteracts endoplasmic reticulum (ER) stress and protects against obesity-induced IR^8^. AMPK is not only a redox-sensitive protein, but is also involved in the response to oxidative stress. Indeed, AMPK was reported to phosphorylate nuclear factor erythroid 2-related factor 2 (Nrf2), a master transcriptional regulator of antioxidant gene programs, which resulted in nuclear accumulation of Nrf2 and subsequent expression of antioxidant genes. Moreover, AMPK can indirectly mitigate reactive oxygen species (ROS) by maintaining high levels of NADPH and GSH (which are cellular antioxidants) through inhibition of fatty acid synthesis and fatty acid oxidation^21^.

Several studies have shown that there is a close link between the dysregulation of AMPK and IR^23^^,^^26–29^. Metformin, as an AMPK activator, has the ability to reduce hepatic glucose production and enhance peripheral insulin sensitivity, in fact it has been used as a first-line antidiabetic agent. Activators of AMPK are considered useful for the treatment of metabolic disorders, and have been the focus of active research and development in the pharmaceutical industry. Therefore, activation of AMPK and related signalling pathways play a vital role in decreasing IR and increasing insulin sensitivity.

### Free fatty acids

There is a certain correlation between obesity and IR, and the increase of circulating FFAs can induce IR through a variety of pathways. Lipotoxicity is also a major factor contributing to the development of IR^30^. Due to saturation of adipose tissue storage capacity, elevated FFAs disrupt cellular homeostasis, resulting in impaired metabolic pathways in surrounding organs such as muscle, liver, and pancreatic tissue. Elevated cyclic FFAs induce the activation of c-Jun N-terminal kinase (JNK), inhibitor of kappa B kinase (IKK) and protein kinase C (PKC), IRS1 phosphorylation^3^. The direct involvement of JNK in insulin signalling is through phosphorylation at Ser307 of IRS1, which inhibits the interaction of IRS1 with insulin receptor and inhibits its tyrosine phosphorylation^4^. In addition, the interaction of PKC, JNK and IKK disrupts metabolic homeostasis by activating kinases involved in IRS serine phosphorylation and inhibits insulin action^3^^,^^13^^,^^15^. These kinases also activate nuclear factor-κ B (NF-κB) signalling, which leads to a sudden inflammatory response. The lipid metabolite diacylglyceride (DAG) has also been shown to induce IR. Increased muscle DAG (intramyocellular lipid) contributes to muscle IR by activating PKC-θ and inducing IRS1 Ser-307 phosphorylation. Ceramide has been shown to induce IR through PKC and JNK activation. Ceramide also inhibits Akt activation by increasing PPAR interaction with Akt and phosphorylates the Thr-34 site of Akt by PKC, resulting in reduced PIP3 binding to Akt^8^. So, targeting hepatic lipid metabolism have beneficial effects on IR, such as a ketohexokinase inhibitor or a protein tyrosine phosphatase-1B(PTP1B) inhibitor.

### Inflammation and oxidative stress

Chronic low-level inflammation caused by obesity is a key factor in obesity-related IR. Adipose tissue expansion is a response to heat overload and is associated with increased immune cell infiltration and subsequent proinflammatory response. In this case, two cell types are particularly important: adipocytes and macrophages, both of which are able to secrete proinflammatory cytokines and induce IR^5^^,^^6^. Increases in monocyte chemoattractant protein-1 (MCP-1), a chemokine secreted by adipocytes, prompts macrophage accumulation into adipose tissue and induce IR. Shimobayashi et al.^29^ found that IR in adipocytes leads to the production of MCP-1, which recruits monocytes and activates pro-inflammatory macrophages. Finally, IR was associated with reduced insulin/mTORC2 signalling and increased MCP-1 production in the visceral adipose tissue of obese subjects. In obesity, cytokines secreted by immune cells and adipocytes such as tumour necrosis factor-α (TNF-α), interleukin-1 (IL-1) or IL-6, are increased and induce IR through multiple mechanisms, including activation of serine/threonine kinases, reduction of IRS1, GLUT4 and PPAR-γ expression or activation of suppressor of cytokine signalling 3 in adipocytes^3^^,^^23^^,^^27^. Another driver of obesity-related inflammation is the activation of toll-like receptors (TLRs), especially TLR-2 and TLR-4. TLRs belong to the innate immune system, are usually activated by pathogen-associated molecular patterns (e.g. lipopolysaccharide, LPS), and induce inflammation by activating the NF-κB pathway^13^. TLRs are commonly expressed in skeletal muscle and adipose tissue of obese patients, especially the elevated level of TLR-4. Interestingly, saturated FFAs can also activate the pathway, suggesting a possible role for these receptors in obesity-driven inflammation^3^^,^^7^. Thus, reduced TLR-2 or TLR-4 signalling proteins could protect them from obesity and obesity-related IR. There is low-grade inflammation affecting all organs in patients with type 1 and type 2 diabetes. Zhao et al.^31^ found that inflammation-induced microvascular IR is an early event in diet-induced obesity.

Oxidative stress is the result of ER stress caused by ROS overproduction in mitochondria. Under normal physiological conditions, free radical levels and antioxidant defences are in balance. In the case of over-nutrition, mitochondria are hyperactive and produce more reactive oxygen species. Redox imbalance occurs when the generation of reactive oxygen species molecules exceeds their scavenging capacity. Oxidative stress caused by ROS can form a vicious cycle, further damaging the cellular infrastructure and promoting IR^15^. In obese and diabetic states, an imbalance between antioxidant defense systems and pro-oxidants occurs, and ROS levels are elevated, which may be due to increased flux of metabolites into mitochondria, altering mitochondrial proteins and reducing expression of antioxidant enzymes^32^^,^^33^. Redox imbalance can cause defects in Nrf2 dependent signalling and impair the antioxidant capacity of the pancreas, which leads to cells becoming very vulnerable to various insults. The transcription factor Nrf2, together with its negative regulator, Kelch-like ECH-associated protein 1 (Keap1), is considered one of the most important cellular defense mechanisms to combat oxidative stress with a particular role in the regulation of antioxidant status^34^. Many endogenous enzymes are downstream targets of Nrf2 and catalyse antioxidant reactions, such as glutathione peroxidase (GPx), superoxide dismutase (SOD), haem oxygenase-1 (HO-1), NADPH-quinone oxidoreductase-1 (NQO-1), and glutathione-S-transferase (GST). Among the protein kinases downstream of Akt, glycogen synthase kinase 3β (GSK-3β) was found to be capable of directly phosphorylating Nrf2^34^^,^^35^.

Increased oxidative stress leads to activation of stress kinases that induce IR through serine phosphorylation of IRS proteins^3^. In addition to ROS-mediated aspects of IR, alterations in mitochondrial dynamics cause IR in the form of increased mitochondrial fission and can be rescued by inhibiting fission, thereby reducing p38 MAPK activity and increasing IRS1 and Akt activation^13^. The effects of mitochondrial perturbations can lead to down-regulation of key regulators of mitochondrial biogenesis PGC-1α and increase mitochondrial apoptosis sensitivity^22^. Mitochondrial sirtuins, sirtuin3-5, respond to nutrient availability and oxidative stress to modulate cellular metabolic homeostasis^36^. Hepatic mitochondrial FA oxidative damage also leads to elevated DAG content, resulting in activation of PKC-1, decreased IRS2 phosphorylation and PI3K activity. Excessive ROS will directly stimulate IKK/NF-κB and JNK pathways^3^^,^^4^^,^^13^. The chronic elevation of FFAs is associated with inflammation through activation of PKC, IKK/NF-κB, JNK pathway, endoplasmic reticulum stress, redox imbalance and other pathways, resulting in dysregulated intracellular signal transduction and ultimately leading to pathological conditions of IR^29^^,^^31^.

In the Maillard reaction, reducing sugars react nonenzymatically with amino groups of proteins, lipids, and nucleic acids to generate AGEs. AGEs production is primarily endogenous and can be induced to form aggregated proteins. AGE-mediated damage occurs through interaction with the cell surface pattern recognition receptor (RAGE). RAGE is commonly expressed in a variety of cell types and plays a vital role in the development of pancreatic β-cell dysfunction and the prognosis of diabetes. Chronic activation of RAGE ligands contributes to the upregulation and activation of NF-κB, a sequence-specific master transcription factor^3^. NF-κB belongs to the Rel family proteins and plays a vital role in inflammation associated with IR. Following NF-κB activation, expression of RAGE and pro-inflammatory cytokines such as TNF-α, IL-1β, and IL-6 begins to increase, which in turn triggers ER stress^8^. ER stress, once triggered, causes the unfolded protein response to restore cellular homeostasis, thereby increasing intracellular ROS formation^4^^,^^8^. Inflammation, oxidative stress, and PKC activation would inhibit IRS1 activity by enhancing serine/threonine phosphorylation and reducing tyrosine phosphorylation of IRS1, which leads to impaired insulin signalling^8^^,^^9^^,^^37^. In recent years, many studies have verified the activity of anthocyanins against inflammation and oxidative stress^9^^,^^12^^,^^23^^,^^27^. Any measure that reduces inflammation, improves oxidative stress, and targets proteins involved in signalling pathways is of great help in reducing IR.

## Flavonoids: Classification and biological properties

Flavonoids are benzo-γ-pyrone derivatives comprising of two phenolic rings and a pyran ring. Arrangement of hydroxyl (-OH), methoxy (-OCH_3_), and glycosidic side groups and conjugations among the rings A and B make different pharmacological effects. Polymerisation of this nuclear structure yields tannins and other composite species that provide help for its antioxidant activity^38^. The substitution of hydroxyl and methoxy groups plays a vital role in reduction of IR. Due to their potent antioxidant and anti-inflammatory properties, flavonoid-rich diets are promoted for maintaining good health and well-being and prevention of diabetes, obesity, cardio-vascular diseases, and neurodegenerative disorders. The ability of flavonoids to decrease IR lies not only in the interaction with insulin-related signalling pathway, but also in the antioxidant and anti-inflammatory biological activities of flavonoids. Numerous scientific evidences have shown that flavonoids can participate in glucose metabolism-related pathways. Over the years, the effects of flavonoids on the insulin-signalling pathway have been widely assessed in *in vitro* experiments (Table 1) and in vivo models (Table 2).

**Table 1.** Effects of flavonoids on decreasing IR in *in vitro* experiments.

Flavonoids	Quantity	In vitro Model	Activity	Effect	References
Luteolin	20 μM	3T3-L1 adipocytes and primary adipose cells	↓TNF-α, IL-6, MCP-1 ↑PPAR-γ, GLUT4, p-Akt2	Increase adiponectin and leptin, increase the response of glucose uptake	^39^
	20 μM	a coculture system of adipocytes and macrophages cells	↓JNK, TNF-α, MCP-1, NO	Suppress the production of inflammatory mediators, attenuate IR	^40^
Apigenin	25 μM	HepG2 Cells	↓SREBP-1C, SREBP-2, ER stress	Inhibit ER stress and decrease blood lipid and IR	^11^
Nobiletin	10 μM	C3A liver cells	↑IRS1, PI3k, GLUT2 ↑AMPK	Alleviate dyslipidaemia and IR	^41^
Chrysin	10–15 μM	HepG2 Cells	↑AMPK ↑PI3K, p-IRS1, p-Akt	Decrease IR, oxidative stress, inflammation and liver injury	^42^
Quercetin	10 μM	HepG2 Cells	↓TNF-α, IL-8 ↑SOD, CAT, GSH-Px	Decrease triacyl glycerol accumulation, IR, inflammatory cytokine secretion increase cellular antioxidants	^43^
Kaempferol	10–50 μM	HepG2 Cells	↑Akt, hexokinase ↓PC	Suppress glucose production, improve insulin sensitivity	^44^
Rutin	100 μM	Myotubes cells	↑IRk, GLUT4	Increase glucose uptake, decrease IR	^45^
Daidzein	25 μM	Adipocyte and Macrophage Co-Cultures	↑PPAR-α, PPAR-γ ↓NF-κB, JNK	Decrease chronic inflammation, alleviate IR	^46^
	100 μM	L6 myocytes	↑GLUT4, AMPK	Promote glucose uptake, suppress serum total cholesterol levels	^47^
Naringin	5 μM	HepG2 cells	↓RAGE, AGEs, NF-κB, ROS	Mitigate inflammation, oxidative stress and mitochondrial apoptosis	^48^
Hesperidin	10 μM	L6 myotubes	↓ROS, AGEs, PI3K ↑GLUT4, IRS, Akt	Improve insulin sensitivity, reduce oxidative stress response	^49^ ^,^ ^50^
	25 μM	HepG2 cells	↑IRS1, GLUT2 ↓TLR4, NF – κB	Increase glucose uptake, decrease IR	^51^
EGCG	10–100 μM	HepG2 cells, 3T3-L1 adipocytes	↑GLUT2, PGC-1b ↓SREBP-1c, FAS ↓NF-κB, TNF-α, IL-6, ROS, MDA, p53, TNF-α	Increase glucose uptake, decrease IR	^52–54^
	50 μM	HepG2 cells	↓PTP1B ↑IRS1, Akt, GLUT2	Accelerate the glucose uptake, ameliorate metabolic misalignment	^55^
Anthocyanins	10–20 ug/mL	3T3‐L1 adipocytes	↓NF – κB ↑PI3K, p-Akt, GLUT1	Decrease adipocyte inflammation, IR	^56^
	50 μM	3T3‐L1 adipocytes	↑p-Akt, p-IKK, p-MEK ↑GLUT4	Reduce hyperglycaemia, ameliorate inflammation and IR	^57^

**Table 2.** Effects of flavonoids on decreasing IR in in vivo experiments.

Flavonoids	Quantity/Day	Animal Models/Length of Study	Activity	Effect	References
Luteolin	20–100 mg/kg	High-fat fed rats/12 weeks	↓IL-6, TNF-α, MCP-1 ↓PKC	Decrease glucose intolerance and insulin sensitivity reduce body weight gain, fat deposition, and adipocyte hypertrophy	^58^
	50–100 mg/kg	High-fat fed rats/16 weeks	↓SREBP1, FFA ↑IRS2, PPAR-γ	Reduce hepatic lipid load, improve chronic low-grade inflammation, increase insulin levels, alleviate IR	^59–61^
	100 mg/kg	High-fat fed rats/20 weeks	↑AMPKα ↑M1 polarisation	Inhibit inflammatory polarisation, enhance insulin signals	^62^
Apigenin	200 mg/kg	High-fat fed mice/12 weeks	↑p-AMPK, p-ACC ↑UCP-1, PGC-1α ↓NF-кB, MAPK ↓FFAs	Ameliorate body weight gain, glucose intolerance and IR enhance lipid catabolism, thermogenesis and browning	^63^
	10 mg/kg	High-fat fed rats/12 weeks	↓SREBP-1C, SREBP-2, ER stress	Inhibit ER stress and decrease blood lipid and IR	^11^
	10–50 mg/kg	High-fat fed mice/16 weeks	↑PPAR-γ	Improve glucose resistance, reduce liver and muscular steatosis	^64^
	50 mg/kg	High-fructose fed mice/4 weeks	↑HO-1, NQO1	Decrease IR, dyslipidemia and liver injury	^65^
Nobiletin	10–100 mg/kg	High-fat fed rats/8 weeks	↑PPAR-γ, PAR-α, SREBP-1C ↓TNF-α, MCP-1 ↑GLUT4, p-Akt	Improve plasma adiponectin levels and glucose tolerance, improve dyslipidemia, decrease hyperglycaemia and IR	^66^ ^,^ ^67^
Scutellarein	50 mg/kg	High-fat fed rats/16 weeks	↑AMPK ↑p-IRS1, p-Akt	Weight loss, improve glycometabolism, against oxidative stress	^68^
Chrysin	30 mg/kg	High-fat fed mice /STZ-induced Mice/5 weeks	↑AMPK ↑PI3K, p-IRS1, p-Akt	Decrease IR, oxidative stress, inflammation and liver injury	^42^
Quercetin	30 mg/kg	High-fat fed rats/6 weeks	↓NF-κB, TNF-α, IL-6, TLR-4 ↓SREBP-1c, FOXA1	Reduce HOMA-IR, inhibit inflammasome response and net stress, reduce hepatic lipid accumulation	^69^ ^,^ ^70^
	50 mg/kg	High-fat fed mice/6 weeks	↑p-Akt, IRS1, GLUT4 ↓NLRP3	Reduce inflammation, IR and HOMA-IR, improve insulin sensitivity	^28^ ^,^ ^71^
Kaempferol	50–150 mg/kg	High-fat fed rats/10 weeks	↓NF-κB, TNF-α, IL-6, IKK	Inhibit inflammatory response, ameliorate IR	^72^ ^,^ ^73^
	100 mg/kg	High-fat fed Mice/20 weeks	↑GLUT4, AMPK	Improve hyperglycaemia, hyperinsulinemia, and circulating lipid profile. Preserved Pancreatic *β*-Cell Mass	^74^
Fisetin	200 mg/kg	High-fat fed rats/16 weeks	↓GKPEPCK, G6Pase ↑IRS2	Prevent adiposity, ameliorate IR, normalise pancreatic islet dysfunction, decline hepatic gluconeogenesis and proinflammatory responses	^75^
Daidzein	100 mg/kg	db/db mice/5 weeks	↑GLUT4, AMPK	Promote glucose uptake, suppress serum total cholesterol levels	^47^
	50 mg/kg	ovariectomized rats/12 weeks	↓TNF-α, IL-6	Decrease weight gain, visceral fat and HOMA-IR	^76^
Genistein	10–20 mg/kg	Diabetic rats/4 weeks	↓LDL, VLDL, TC, TG	Decrease HOMA-IR, enhance beta cell function and decrease IR	^77^
	20–40 mg/kg	High-fat diet/streptozotocin induced mice/8 weeks	↓LPS, TNF-α, IL-1β, IL-6, NLRP3	Decrease HOMA-IR, ameliorates inflammation and IR	^78^
Naringin	100 mg/kg	High-fat fed rats/12 weeks	↓RAGE, AGEs, NF-κB, ROS	Mitigate inflammation, oxidative stress and mitochondrial apoptosis	^48^
	200 mg/kg	High-fat fed rats/20 weeks	↑AMPK, p-IRS1, MAPKs ↓p-JNK, p-NF-κB	Reduce oxidative damage, reduce chronic inflammation and IR	^79^
Hesperidin	100 mg/kg	High-fat fed rats/20 weeks	↑p-insulin receptor, p-PDK1	Improve insulin sensitivity, regulate glycolysis and gluconeogenesis, increase glucose uptake	^80^
EGCG	50 mg/kg	High-fat fed rats/12 weeks	↑Akt, GLUT4, AMPKα, sirtuin1 ↓mTOR, SREBP-1c ↑IRS1, AS160	Increase glucose uptake, decrease IR	^81^
	50 mg/kg	High-fat fed mice/16 weeks	↓PTP1B ↑IRS1, Akt, GLUT2	Accelerate the glucose uptake, ameliorate metabolic misalignment	^55^
Anthocyanins	50–200 mg/day	High-fat fed rats/12 weeks	↓TLR4, NF-κB, JNK ↑Nrf2, HO-1, NQO1 ↑IRS1, Akt	Decrease inflammation and oxidative stress, alleviate IR	^82^

### Flavones

**Figure 4.**
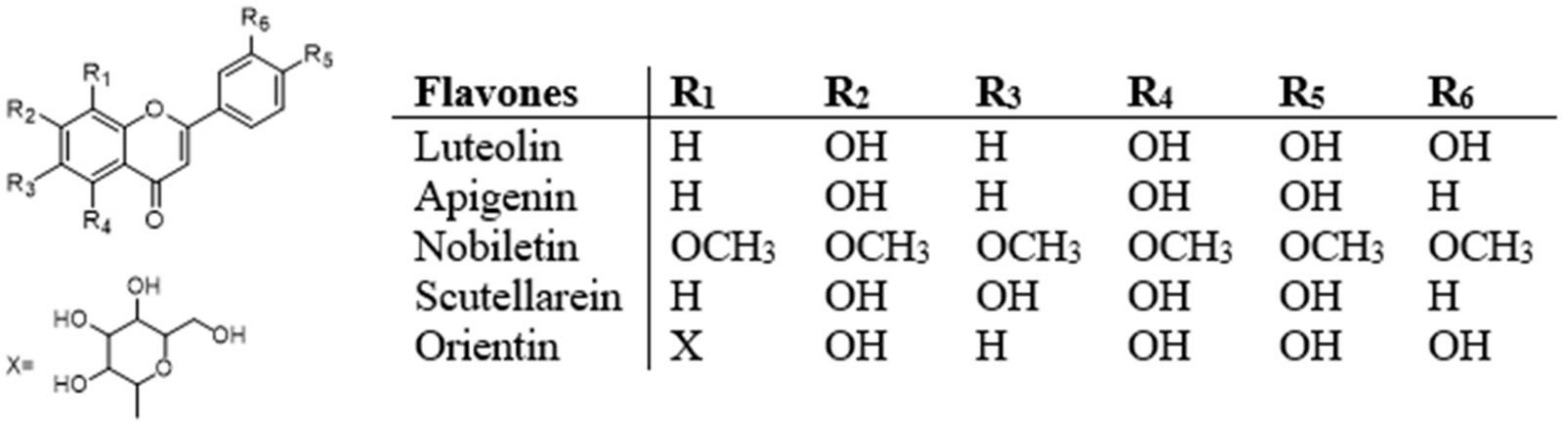
Structures of flavones.

Flavones are found mainly in celery, parsley, and many herbaceous plants (Figure 4)^37^. The most important dietary flavones are apigenin and luteolin. Luteolin is an important component of flavonoids commonly found in vegetables, fruits, traditional Chinese medicinal materials, and other foods and is synthesised in plants through the phenylpropanoid pathway^6^. Studies have found that ingestion of luteolin-rich extracts or isolated luteolin has a variety of health-promoting and pharmacological effects, such as anti-oxidation, liver protection, anti-cancer and neuroprotective function^59^^,^^62^^,^^83^. Accumulating evidence suggests that luteolin intake may be beneficial for disorders of glucose and lipid metabolism, particularly IR, diabetes and obesity. Luteolin can maintain glucose and lipid metabolic balance, decrease IR and manage metabolic syndrome through liver, fat, and skeletal muscle tissue^84^.

In a co-culture system using 3T3-L1 adipocytes and RAW264 macrophages, luteolin (20 μM) was found to inhibit JNK activation and block proinflammatory factor expression (TNF-α, MCP-1 and NO), thereby playing a major role in the regulation of adipose tissue inflammation and IR in obesity^40^. Treatment of 3T3-L1 adipocytes with 1–20 μM luteolin for 6 h enhanced GLUT4-mediated glucose uptake through Akt2 activation of the insulin signalling pathway^39^. Skeletal muscle has been the target of increasing therapeutic intervention, and treatment with *Platycodon grandiflorum* seed extract (0.4 mg/mL, luteolin accounts for 38% of total polyphenols) activates IRS1/PI3K/Akt/AS160 signalling and insulin-independent AMPK in insulin-resistant muscle cells, thereby inducing GLUT4 translocation to the plasma membrane (PM), which may be attributed to the proglucose uptake effect of luteolin^85^. In an in vivo study, Kwon et al.^60^ found that dietary luteolin supplementation protected against diet-induced hepatic steatosis and IR in mice. Compared with high fat diet (HFD), luteolin treatment (HFD with 0.005% luteolin) alters hepatic lipid and glucose metabolism factors, thereby preventing fat accumulation and steatosis, and ultimately decreasing IR by reducing hepatic lipid load. Western blot and RT-qPCR analysis showed that luteolin increased hepatic insulin sensitivity by inhibiting the expression of SREBP1. Also, luteolin decreased IR by increasing the expression of IRS2 and suppressing gluconeogenesis. In addition, luteolin could increase the uptake of FFAs from plasma by adipocytes to activate PPAR-γ, thereby reducing dyslipidemia and improving hepatic insulin sensitivity. Also, Kwon et al.^83^ found that luteolin (HFD with 0.005% luteolin) can prevent IR by targeting TLR signalling and down-regulating the expression of adipocyte genes involved in inflammation, improving chronic low-grade inflammation and further increasing pancreatic β-cells quality and insulin levels. Another in vivo study showed that chrysin (20 mg/kg/day) and luteolin (100 mg/kg/day) could inhibit the occurrence of hyperinsulinemia and the increase of serum AGEs and blood lipids in hyperfructose load, while PPAR-γ antagonist, bisphenol A diglycidyl ether reduced these effects. Therefore, the mechanism of chrysin and luteolin in alleviating IR and related vascular complications may be through activation of PPAR-γ^61^.

Luteolin reduces IR in HFD-induced mice, which may involve activation of AMPK signalling^62^. AMPK is an important nutrient sensor and inflammatory suppressor^3^. In macrophages, the catalytic subunit AMPKα1 mainly suppresses proinflammatory responses and polarisation. Zhang et al.^62^ found that dietary luteolin (HFD with 0.01% luteolin) might suppress inflammatory adipose tissue macrophage (ATM) polarisation by activating AMPKα1 signalling. Specifically, luteolin counteracted LPS- and PIG (Palmitate insulin and glucose)-induced overexpression of the proinflammatory cytokine genes Mcp1, Tnf-α and Il-6 in RAW264.7 cells and peritoneal cavity resident macrophages (PCMs). Also, polarisation-associated alterations in macrophage markers, including the M1 marker Nos2, the M2 marker Arg1 and the metabolic activation macrophage markers Cd36 and Plin2 were also turned back by luteolin treatment. However, the improvements in IR might not be solely attributed to the inhibition of ATM polarisation and inflammation by luteolin in HFD-fed mice^86^. In addition, as an important nutrient sensor, AMPK can promote fatty acid oxidation and energy expenditure. Indeed, dietary luteolin not only suppressed epididymal adipose tissue macrophage polarisation, but also reduced body and subcutaneous adipose tissue weights^62^^,^^86^. In addition, luteolin inhibits several important pro-inflammatory cytokines related to IR, including TNF-α, IL-6 and MCP-1, and decreases IR by alleviating inflammatory response^58^. Baek et al.^59^ found that loss of ovarian function in HFD-mice increased IR and adipose tissue inflammation, and luteolin (HFD with 0.005% luteolin) had a protective effect. This effect may be achieved by inhibiting M1-like polarisation of macrophages in adipose tissue.

Apigenin is a natural plant flavonoid, widely distributed in the plant kingdom, exists in many fruits, vegetables, herbs and spices^11^. Apigenin is biosynthesized by the phenylpropanine pathway. Many studies have shown that apigenin has a variety of biological functions, such as anti-proliferation, anti-inflammation, anti-obesity, anti-oxidation and anti-cancer properties^87^. There are also many reports on apigenin decreasing IR and improving metabolic syndrome. Apigenin (10–50 mg/kg/day) can increase the mRNA expression of adipose triglyceride lipase (ATGL), hormone-sensitive lipase (HSL), FoxO1 and sirtuin1 in HFD mice, and ATGL and HSL are mainly responsible for fat decomposition. Moreover, SIRT1 controls adipocyte decomposition through FoxO1-mediated ATGL expression^64^^,^^68^^,^^87^. Excessive liposolysis leads to high levels of circulating FFAs, inducing deposition of triglyceride (TG) in non-adipose tissue. Sun et al.^63^ also found that dietary apigenin (200 mg/kg/day) increased the expression of p-AMPK and p-ACC in adipose tissue, resulting in fatty acid oxidation, without increasing FFAs and TG. Apigenin also enhances uncoupling protein-1 (UCP-1) and PGC-1α in SAT and BAT to consume plasma free fatty acids, thereby decreasing obesity-related IR. In HFD mouse model, Wu et al.^11^ also found that apigenin (10 mg/kg/day) inhibited ER stress and decreased blood lipid and IR by down-regulating SREBP-1c, SREBP-2 and their downstream genes.

Feng et al.^64^ found that apigenin (10–50 mg/kg/day) activates PPAR-γ in HFD and ob/ob mice, inhibits NF-κB activation by blocking the translocation of p65/PPAR-γ complex to the nucleus, and reduces proinflammatory cytokine levels, further reducing inflammation and IR. Sun et al.^63^ also found that apigenin (200 mg/kg/day) decreases IR by inhibiting inflammation of adipose tissue and promoting fat decomposition via MAPK and NF-кB signalling pathways. In animal models of high fructose feeding for 4 weeks, Yang et al.^65^ found that apigenin (50 mg/kg/day) plays a beneficial role by decreasing IR, alleviating liver damage, and inhibiting lipid profile changes. Also, apigenin may inhibit Nrf2 nuclear translocation by interfering the binding of Keap1 to Nrf2, thus increasing the expression of antioxidant genes such as HO-1 and NQO-1.

Long-term dietary supplementation of nobiletin (10–100 mg/kg/day) improves liver steatosis, inflammation, and IR by activating PPAR-γ, regulating the expression of genes related to lipid metabolism, and increasing the activity of insulin signalling pathways^66^^,^^67^. Gao et al.^68^ found that scutellarein (50 mg/kg/day) further alleviates IR through antioxidant stress and activation of AMPKα pathway in a HFD-induced mouse model. In insulin-resistant HepG2 cells, chrysin (10–15 μM) ameliorates glucose and lipid metabolism disorders and IR by modulating the AMPK/PI3K/Akt signalling pathway^42^. Orientin improves substrate utilisation and expression of major genes involved in insulin signal transduction and energy regulation in insulin-resistant hepatocytes. Sithandiwe et al.^41^ found that orientin (10 μM) treatment of insulin-resistant liver cells can combat dyslipidemia or IR. This may be related to orientin improving gene expression of GLUT2, insulin signal transduction (IRS1 and PI3k), and energy regulation (AMPK and Cpt1).

### Flavonols

**Figure 5.**
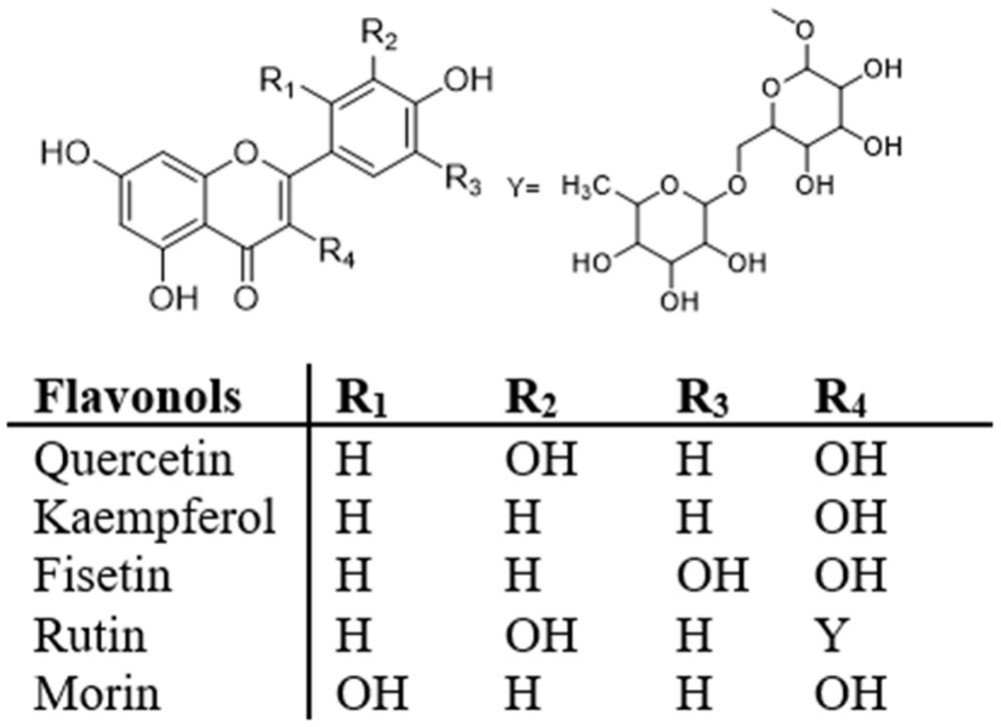
Structures of flavonols.

Flavonols are the most abundant flavonoids in the plant kingdom (Figure 5). The main flavonoid components of the diet are flavonols including quercetin, kaempferol, rutin, isornetin, fisetin and myricetin^88^. Quercetin is an important natural flavonoid compound, which has many functions such as lowering blood pressure, lowering blood lipid, lowering blood sugar, anti-oxidation, anti-virus, anti-cancer, anti-inflammation and nerve protection^88–90^. Quercetin has been the focus of medical attention because of its potential health benefits^89^. In animal models, quercetin can promote insulin secretion, decrease IR, reduce lipid levels, inhibit inflammation and oxidative stress, and reduce liver fat accumulation^89^. Arias et al.^69^ reported that quercetin (30 mg/kg/day) reduced IR in diabetic rats assessed by homeostasis model assessment (HOMA). Porras et al.^70^ found that quercetin (HFD with 0.05% quercetin) reduces hepatic lipid accumulation and thus IR by regulating the expression of lipid metabolism genes, cytochrome P450 dependent lipid peroxides and associated lipid toxicity. Quercetin reverses intestinal microbiota imbalance and related endotoxemia mediated induction of TLR-4 pathway, inhibits inflammasome response and net stress pathway activation, leads to the blocking of dysregulated gene expression of lipid metabolism, and further decreases IR. Vidyashankar et al.^43^ found that quercetin (10 μM) decreased IR and up-regulated cellular antioxidants in oleic acid-induced steatosis of HepG2 cells. RT-PCR results confirmed that quercetin (10 μM) inhibited TNF-α gene expression. Jiang et al.^71^ found that quercetin (50–100 mg/kg/day) reduced PM-induced circulating inflammation and adipose tissue inflammation by inhibiting NOD-like receptor protein 3 (NLRP3) inflammasome, and decreased PM-induced IR by salvaging damaged insulin signal transduction in adipose tissue (up-regulating the levels of insulin signalling molecules, such as p-Akt, IRS1 and GLUT4). Tan et al.^28^ found that compared with the group of HFD alone, quercetin supplementation (HFD with 0.05% quercetin) significantly increased the expression of GLUT4 in mice, reducing HOMA-IR. In skeletal muscle cells (L6 myotubes), quercetin (100 μM) activates the AMPK pathway to correct IR by upregulating AMPK and its downstream target P38 MAPK protein and mRNA levels^91^. Henagan et al.^22^ reported that long-term low-dose (50 μg/day, but not high-dose, 600 μg/day) dietary quercetin supplementation improved skeletal muscle mitochondrial function and attenuated diet-induced IR by up-regulating skeletal muscle PGC-1α.

Kaempferol is a flavonol found in apples, grapes, tomatoes, tea, potatoes, broccoli, spinach and some edible berries^72^. Alkhalidy et al.^74^ reported that dietary intake of kaempferol (0.05% in diet) improved insulin sensitivity in middle-aged obese diabetic mice, thereby improving hyperglycaemia, hyperinsulinemia, and circulating lipid profile. Kaempferol treatment reversed impaired GLUT4 and AMPK expression in HFD in muscle and adipose tissue of obese mice. In another study by Alkhalidy, kaempferol (50 mg/kg/day) decreased IR by over-inhibiting hepatic gluconeogenesis and enhancing hepatic insulin sensitivity in diet-induced obese mice^44^. Luo et al.^72^ found that the anti-inflammatory properties of kaempferol were closely related to the improvement of IR. Western blotting showed that kaempferol (50–150 mg/kg/day) induced the reduction of p-IRS1 (Ser307), p-IKKα and p-IKKβ in a HFD and streptozotocin induced diabetic rat model. These effects were positively accompanied with the reduction in NF-κB, TNF-α and IL-6 levels. These results suggest that kaempferol ameliorates insulin signalling pathway defects in diabetes mellitus by mediating IKK down-regulation and inhibition of NF-κB pathway activation, thereby decreasing IR. Khlif et al.^73^ found that *Saussurea multiflora* extract (250 mg/kg/day) rich in quercetin-3-O-glucoside and kahenol-3-O-glucoside decreased obesity-related IR associated with a reduction in inflammatory pathways. Nie et al.^92^ designed and synthesised new isoxazole derivatives using kaempferol as the lead compound. The derivative improved glucose consumption in insulin resistant HepG2 cells at the nanoscale (EC50 = 0.8 nM) level. Western blotting showed that it significantly increased the phosphorylation of AMPK and decreased the levels of PEPCK and G6Pase in HepG2 cells. Therefore, it activates AMPK/PEPCK/G6Pase pathway, promoting glucose uptake and decreasing IR.

Fisetin is a tetrahydroxy flavonoid found in fruits and vegetables. Choi et al.^75^ reported that fisetin (200 mg/kg/day) protects HFD-induced islet hypertrophy, thereby normalising plasma insulin concentration. Hepatic mRNA expression also showed that fisetin supplementation significantly down-regulated GK PEPCK and G6Pase genes and regulated IRS2 genes in mice. Compared with the HFD group, the fisetin group significantly reduced liver lipid levels by increasing FA oxidation and reducing fat production, which in turn significantly reduced liver fibrosis, which may help prevent IR. Rutin, a glycosylated quercetin, can often be extracted from natural plants such as buckwheat, oranges, grapes, lemons, limes, peaches, and berries, and has also been reported to have anti-obesity and anti-diabetes properties. Hsu et al.^45^ found that rutin (100 μM) enhanced the activity of insulin-dependent receptor kinase (IRK) and the translocation of GLUT4 in differentiated muscle tubes, thereby increasing glucose uptake and decreasing IR. In the rat skeletal muscle L6 cell line model, morin decreased associated IR by decreasing oxidative stress. RT-PCR analysis showed that morin (60 µM) significantly increased the expression of antioxidant genes GPx, GST and insulin signalling pathway genes insulin receptor tyrosine kinases, IRS1, PI3K, GLUT-4 and GSK-3β in L6 myotubes^93^.

### Isoflavones

**Figure 6.**
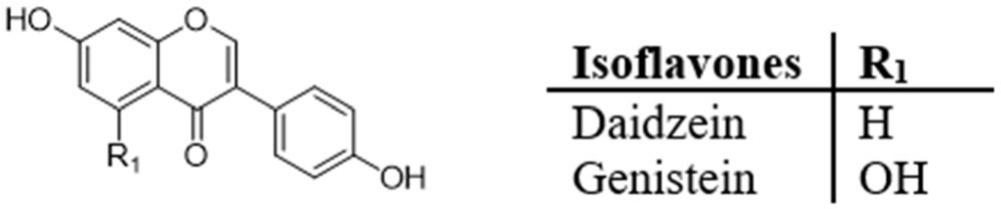
Structures of isoflavones.

Isoflavones (Figure 6) are found mainly in soybeans and other legumes, such as genistein and daidzein^94^. A large number of studies have shown that isoflavones have positive effects on obesity, glucose homeostasis, insulin secretion and lipid metabolism^7^^,^^95^. Daidzein exists mainly in the form of glycosides in *Trifolium pratense*, *Medicago sativa*, *Glycine Max* and some *Leguminosae* plants^96^. Among them, soy and soy products are considered to be the most abundant sources of daidzein^5^^,^^96^. In addition to soybean products, daidzein (glycoside and aglytin form) has also been reported in *pueraria*, a commonly used traditional Chinese medicine^76^. New evidence from epidemiological studies suggests an association between higher soy isoflavone intake and a reduction in T2D and its associated health risks^6^. Several studies have reported the preventive effect of daidzein on improving metabolic diseases, such as hyperglycaemia, IR, dyslipidemia, obesity, and inflammation^96^. Cao et al.^76^ found that daidzein treatment (50 mg/kg/day) can reduce the weight gain, visceral fat, HOMA-IR index and IL-6 levels induced by oophorectomy in mice, decreasing the IR induced by oophorectomy. Sakamoto et al.^46^ verified the mechanism of daidzein (25 μM) in improving chronic inflammation and alleviating IR in a model of 3T3-L1 adipocytes and RAW264 macrophages co-culture. The results showed that daidzein activated PPAR-α and PPAR-γ and inhibited the NF-κB and JNK pathways, thereby regulating the expression of pro-inflammatory genes. In another study, Cheong et al.^47^ found that daidzein (100 μM) improved glucose homeostasis in L6 myoduct cells treated with high glucose. Specifically, the optimal dose of daidzein treatment significantly increased the ratio of GLUT4 to Na+/K + ATPase in the PM of L6 muscle ducts, suggesting a potential role of daidzein in facilitating the translocation of GLUT from intracellular microvesicles to PM. In addition, daidzein supplementation leads to significant phosphorylation of AMPK in high-glucose treated muscle tubes, which in turn promotes GLUT4 translocation and subsequent glucose uptake.

Genistein has beneficial effects on the regulation of glucose homeostasis and management of metabolic syndrome^97^. The only structural difference between these two compounds is the hydroxyl (genistein) or hydrogen (daidzein) moiety at the R1 position. Incir et al.^98^ found that genistein alleviates fructose-induced IR, oxidative stress and inflammation. Specifically, male SD rats aged 6 to 8 weeks supplemented with 20% fructose and 0.25 mg/kg/day genistein significantly reduced HOMA-IR, low-density lipoprotein cholesterol and serum 8-isoprostaglandin levels after genistein administration. In addition, the levels of TNF-α in liver, serum and liver IL-6 and serum visfatin were significantly decreased by genistein. Amanat et al.^99^ reported the effects of genistein on IR, inflammatory factors and lipids in rats with polycystic ovary syndrome. Biochemical and histopathological analyses showed that genistein (20 mg/kg/day) significantly alleviated oxidation, inflammation, blood glucose and histopathological indexes in polycystic ovary syndrome rats.

In HFD/streptozotocin induced C57BL/6J mice, treatment with genistein reduced the levels of hyperglycaemia, hyperlipidaemia and serum proinflammatory factors, and prevented lipid accumulation and IR^77^^,^^78^. Arunkumar et al.^100^ found that genistein (1 mg/kg/day) improved insulin signal transduction, reduced liver fat accumulation and alleviated IR by inhibiting p70S6K. Specifically, genistein activated insulin-stimulated tyrosine phosphorylation of IRS1 and IRS2, PI3K associated with IRS-1/2 and Akt Ser473 phosphorylation, and promoted AMPK Thr172 phosphorylation and inhibited S6K1 by Thr389 phosphorylation in the liver.

### Flavanones

**Figure 7.**
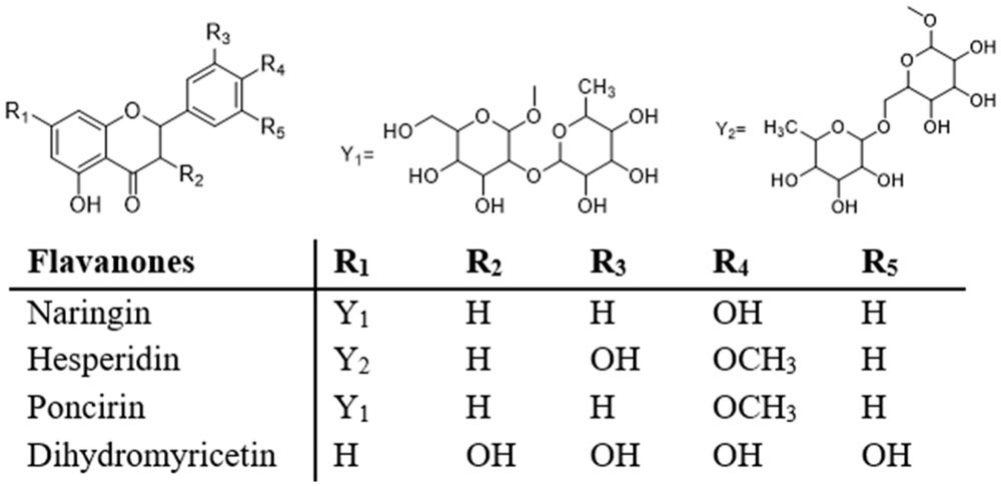
Structures of flavanones.

Naringin and hesperidin are the two main flavonoids found in citrus fruits (Figure 7), such as grapefruits and oranges^6^. Naringin and hesperidin have been reported to have antioxidant, anti-diabetic, lipid-lowering, anti-atherosclerotic and anti-inflammatory activities^5^^,^^6^^,^^95^. Also, naringin has been extensively studied in recent years. Syed et al.^48^ found that naringin (100 mg/kg/day) could reduce the rate of body weight gain, inhibit the expression of RAGE and NF-κB, and improve insulin sensitivity in HFD mice. In an in vivo study, Pu et al.^79^ demonstrated that naringin may have beneficial effects on metabolic syndrome in vivo and *in vitro*. Long-term use of naringin (200 mg/kg/day) enhances liver AMPK activation by promoting AMPK phosphorylation. Activated AMPK ameliorates IR by phosphorylating IRS1 and decreasing phosphorylation of JNK and NF-κB. Meanwhile, activated AMPK protects mice exposed to a HFD from metabolic syndrome by eliminating inflammation and oxidative stress through the MAPKs signalling pathway^79^. Wang et al.^101^ reported a study of naringin improving neuro-insulin signalling and cognitive function in obese mice. The results showed that naringin (100 mg/kg/day) activation of AMPK enhanced the insulin signalling pathway, increased the expression levels of IRS1 and p-Akt in the hippocampus of mice, and improved mitochondrial dysfunction. Fidelix et al.^102^ found in a controlled clinical trial that an orange juice diet alleviates IR, improves blood glucose and lipids and regulates gut microbiota.

Hesperidin has attracted the attention of many researchers for its antioxidant and anti-diabetes potential^103^. In an *in vitro* study of L6 myotube, hesperidin (10 μM) reduced oxidative stress response by scavenging intracellular ROS and inhibiting the formation of AGEs. In addition, naringin down-regulated PI3K activity, whereas up-regulated GLUT4, IRS and Akt expression in L6 myotube, and improved insulin sensitivity^49^. Tian et al.^50^ found that hesperidin (25–100 μM) can reduce ROS, increase the levels of SOD and GPx, improve the oxidative stress and mitochondrial dysfunction induced by high glucose, and further reduce IR. Peng et al.^80^ studied the protective effect of hesperidin on type 2 diabetes in rats with IR induced by alloxan and HFD. They showed that hesperidin (100 mg/kg/day) improved fasting glucose and insulin sensitivity without changing fasting insulin levels, preventing IR and diabetes. oral glucose tolerance test (OGTT) results showed that hesperidin treatment also prevented the development of impaired glucose tolerance. Furthermore, hesperidin induced phosphorylation of insulin receptor and PDK1, while reducing G6Pase activity. In insulin-resistant HepG2 cells, hesperidin (25 μM) up-regulated the expression of IRS1 and GLUT2, down-regulated the expression of TLR4 and NF-κB, thereby increasing glucose uptake and decreasing IR^51^. Rehman et al.^104^ verified the therapeutic effect of hesperidin through protein ligand docking and molecular dynamics simulation. It was found that hesperidin binds to leptin receptor with high affinity and causes a favourable geometric conformation change of leptin receptor, promoting its binding to leptin, which may lead to the final lipolysis and fat cascade, and may eventually lead to the treatment of obesity. At the same time, they found that hesperidin (55 mg/kg/day) controlled levels of altered biomarkers, including glucose, leptin, and IR, in HDF mice at random and fasting states.

Poncirin, a natural flavonoid glycoside, is abundantly found in many citrus fruits. In the insulin-resistant C2 C12 skeletal muscle cell model, western blot analysis showed that poncirin (10 and 15 μM) activated the downstream PI3K/Akt/GSK-3β signalling pathway, improved the expression level of GLUT4, and enhanced insulin sensitivity in insulin-resistant C2 C12 muscle cells by enhancing the phosphorylation of IRS1^105^. He et al.^106^ found that dihydromyricetin (DHM) treatment (100–500 mg/kg/day) increased IRS1 Tyr612 protein phosphorylation levels in liver tissue, indicating that DHM restored impaired insulin signalling pathways and decreased IR.

### Flavan-3-ols

**Figure 8.**
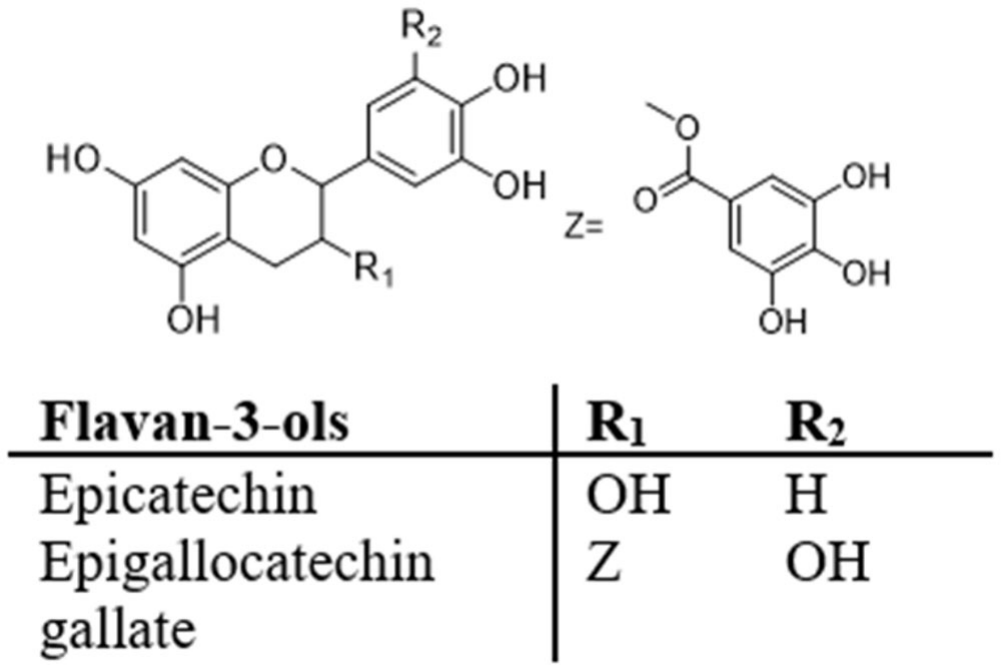
Structures of flavanones.

Flavane-3-ols, also known as flavanols, are found in many fruits, tea, cocoa and chocolate (Figure 8). In fruits and cocoa, the most common falavan-3-ols are catechin and epicatechin, while epicatechin gallate (ECG), epigallocatechin (EGC) and epigallocatechin gallate (EGCG) are found in tea, grapes and certain legumes^107^. Cremonini et al.^108^ found that epicatechin (2–20 mg/kg/day) prevented HFD-induced ileal NADPH oxidase 1/4 upregulation, protein oxidation, and activation of redox-sensitive NF-κB and extracellular regulated protein kinases 1/2 (ERK 1/2) pathways, thereby reducing obesity, steatosis and IR in mice. EGCG, the most abundant catechin in green tea, has anti-inflammatory, antioxidant, hypolipidemia, anti-hyperglycaemia and other biological activities^7^^,^^9^^,^^107^. Zhang et al.^52^ reported that EGCG ameliorates IR in HepG2 cells through the GLUT2/PGC-1β/SREBP-1c/FAS pathway. Specifically, EGCG increases the expression of GLUT2 protein and its downstream PGC-1β in HepG2 cells treated with 25 mM glucose, 0.25 mM palmitic acid (PA) and 50 μM EGCG for 24h. The levels of SREBP-1c and FAS were down-regulated. Meanwhile, EGCG reduces inflammation and oxidative stress-related factors (NF-κB, TNF-α, IL-6, ROS, malondialdehyde MDA and p53) in HepG2 cells and increases glucose uptake, thereby decreasing IR. There is a parallel relationship between chronic low-grade inflammation and IR. EGCG is dose-dependent in decreasing levels of inflammatory mediators and cytokines (IKKβ, p-NF-κB, TNF-α, and IL-6). At the same time, EGCG (10–100 μM) suppresses TLR4 signalling in adipocytes via the 67 kDa laminin receptor and attenuates insulin-stimulated glucose uptake associated with decreased GLUT4 expression^53^. Liu et al.^109^ found that EGCG (3.2 g EGCG/kg chow diet) can restore Akt activity and GLUT4 expression in skeletal muscle of senescence accelerated mice, and activate AMPKα, thereby regulating glucose homeostasis and decreasing IR. In another study, flavanol-rich lychee extracts alleviated IR in obese mice. In skeletal muscle, EGCG (50 mg/kg/day) activates AMPKα and enhances expression of sirtuin1, p-IRS1 and AS160, thereby reducing IR induced by HFD^81^. Ma et al.^110^ found that EGCG (10 μM) affected IR in human HepG2 cells treated with high glucose. Western blot results showed that EGCG restored Akt and GSK levels and increased serine phosphorylation of JNK and IRS1 in HepG2 cells and primary hepatocytes exposed to high glucose. EGCG reduced ROS production induced by high glucose, further decreasing IR.

Mi et al.^54^^,^^55^^,^^111^^,^^112^ conducted several studies on the mechanism of EGCG decreasing IR. In HepG2 cells, they found that EGCG (10–50 μM) eliminates oxidative stress and alleviates IR by enhancing tyrosine phosphorylation of IRS1, stimulating translocation of GLUT2, and activating PI3K/Akt and AMPK signalling pathways. Similarly, in vivo, EGCG (2 g/day) attenuates PTP1B expression, promotes glucose uptake, and activates IRS1/Akt/GLUT2 signalling pathways. Therefore, EGCG is a promising preventive drug against obesity and IR. EGCG (2 g/day) upregulates IRS1/Akt and ERK/CREB/BDNF (brain-derived neurotrophic factor) signalling pathways, significantly decreasing IR and cognitive impairment in HFD and high-fructose-induced mouse models of cognitive impairment. In addition, they found that EGCG (20 μM) alleviates intracellular lipid accumulation, induces phosphorylation of AMPK and ACC, and eliminates redox imbalance to attenuate TNF-α-induced insulin signalling pathway blocking.

### Anthocyanins and other flavonoids

Anthocyanins are found in the human diet in apples, berries, red grapes, eggplant, red cabbage and radishes^23^. More and more studies have been showing that anthocyanins have anti-inflammatory, antioxidant, anti-obesity and anti-diabetes effects^7^^,^^9^^,^^23^. Compared with other flavonoids, anthocyanins have a higher dietary consumption due to their wide distribution. In a multivariate analysis, Jennings et al.^113^ found that higher anthocyanin and flavonoid intakes were associated with a significant reduction in peripheral IR. Lee et al.^114^ found that anthocyanin-rich blueberries (HFD with 10% blueberry powder) could change the intestinal microbiota of rats fed a HFD, reduce systemic inflammation and decrease IR. Tian et al.^82^ reported that anthocyanins (50–200 mg/kg/day) can decrease inflammation and oxidative stress in HFD mice, thus alleviating IR. Specifically, anthocyanins reduce TLR4/NF-κB/JNK in liver tissue and activate Nrf2/HO-1/NQO1. Anthocyanin prevention of HFD-induced gluconeogenesis and IR may be related to its activation of IRS1/Akt. In another HFD-induced mouse model, anthocyanin (200 mg/kg/day) supplementation reduced OGTT and plasma LPS levels, reduced weight gain, improved insulin sensitivity and hyperinsulinemia^115^. Muscara et al.^56^ demonstrated anthocyanins’ role in decreasing IR in 3T3‐L1 adipocytes. Results showed that anthocyanins (10–20 µg/mL) can reverse the NF-κB pathway activated by PA, which inhibits the expression of PI3K/p-Akt axis and GLUT-1 proteins, thereby protecting adipocyte from inflammation and IR. Anthocyanins (50 µM) in purple maize peel increased phosphorylation of Akt, IKK and MEK in 3T3-L1 adipocytes, enhanced GLUT4 membrane translocation, and decreased IR in adipocytes^57^. Daver et al.^12^ found that dietary supplementation with anthocyanins (40 mg/kg/day) was able to inhibit oxidative stress, NF-κB and JNK activation, and PTP1B overexpression. Specifically, anthocyanins reduced phosphorylation of the redox-sensitive signals JNK (Thr183/Tyr185) and IKK (Ser176/180). Therefore, the alleviation of inflammation, oxidative stress and NF-κB/JNK activation are important targets for the beneficial effects of anthocyanins.

Also, two anthocyanidins were also demonstrated to inhibit human carbonic anhydrase VA isoform^116^. Carbonic anhydrase VA is a mitochondrial enzyme which is directly associated with the glucose homeostasis and considered as a promising target for obesity and other associated diseases in humans^117^. Mollica et al.^116^ studied the inhibitory effects of bioactive compounds (anthocyanins and chlorogenic acid) (1 mg/mL) on carbonic anhydrase in blueberry extracts from 14 different Italian cultivars. This could be an innovative target for the development of new inhibitors to reduce insulin resistance. Xu et al.^118^ isolated nine biflavonoids from the ethanolic extract of *Selaginella moellendorffii* and determined their structures by spectral analysis; in insulin-resistant HepG2 cells, the cellular glucose uptake activity was enhanced after biflavonoid treatment (10 µM). However, Western blot analysis showed that one of these compounds dose-dependently increased the expression of p-IRS1, p-PI3K and p-Akt in HepG2 cells without affecting the expression levels of total IRS1, PI3K and Akt, which in turn enhanced insulin sensitivity and decreased IR.

## Potential clinical impact, future perspectives and limitations

The potential clinical uses of flavonoids have been evaluated for different diseases such as diabetes, cardiovascular disease, cancer and Alzheimer. Additional experimental and clinical studies are needed to establish the real therapeutic impact of flavonoids in the near future. Results obtained in *in vitro* and in vivo studies strongly indicate that flavonoids improve insulin sensitivity, increase glucose tolerance and therefore may exert a beneficial effect on IR. Currently, some clinical trials have assessed the effects of flavonoid-rich food intake on IR (Table 3). The results of A Comparison Chocolate with and Without High Cocoa Solids in Patients with Type 2 Diabetes in a Randomised Clinical (NCT01617603) have been reported. The trial compared differences in IR (HOMA) between treatments after 12 weeks of product intake. Average HOMA decreased from 6.50 to 2.81 in the high-polyphenol milk chocolate (approximately 1 mg/g with epicatechin) + cocoa polyphenols (20 g/day, two active products provide 20 mg/day epicatechin) group. Average HOMA decreased from 4.63 to 2.21 in the low polyphenol milk control (approximately 0.05 mg/g with epicatechin) + cocoa polyphenols (20 g/day, two active products provide 20 mg/day epicatechin) group. Although the trial examined both endothelial function and oxidative stress in patients with type 2 diabetes, ClinicalTrials.gov did not report measurements. According to HOMA values, the intake of epicatechin-containing foods can reduce IR in patients with type 2 diabetes.

**Table 3.** Current clinical trials on flavonoids as potential therapy against IR.

Clinical Trial Identifier No.	Objective	Voluntary and Dose	Status
NCT04306406	Molecular Mechanisms of Raspberries Effect on IR and Inflammation (RASPBERRY)	9 male and female of 18–70 age were given single serving smoothies drink made with red raspberries to be consumed daily for two weeks	Completed
NCT01350843	The Effects of Orange Juice on Plasma Lipids	36 males of 40–60 age were given 250 mL of orange juice or a sugar matched orange drink daily	Completed
NCT02728570	Effect of Dietary Flavonoids on Intestinal Microbiota, Intestinal Inflammation and Metabolic Syndrome	30 male and female of 18–70 age were given a prepared diet with Low Dietary Flavonoids (10 mg of flavonoids/1000 Kcals) for six weeks. After a minimum washout period of 2 weeks, participants receive a prepared diet with High Dietary Flavonoids (340 mg of flavonoids/1000 Kcals) for six weeks.	Completed
NCT05158673	Effect of Cocoa Polyphenols Supplementation on Cardiovascular Risk of Postmenopausal Women	30 females of 50–60 age with an early post menopause diagnosis were given two capsules of 500 mg of the flavonoid supplementorally every 12 hours for 12 weeks.	Not yet recruiting
NCT01944579	Blackberry Flavonoid Absorption and Effects on Intestinal Bacteria	46 male and female of 25–75 age were given a controlled diet with the control food (jello) first and then cross over to the controlled diet with blackberries.	Completed
NCT02006810	Development of Endothelial Biomarkers (NUTREND)	50 males of 40–65 age were given a single dose of lipids or a single dose of flavonoids	Completed
NCT04616404	The Metabolic Effects of Cynara Supplementation in Overweight and Obese Class I Subjects with Newly Detected Impaired Fasting Glycaemia.	44 male and female were given tablets containing 500 mg of artichoke extract	Completed
NCT05243238	Hesperidin and Diosmin Effect on Metabolic Syndrome	129 male and female of 18–70 age were given either hesperidin, diosmin, or combination of both	Completed
NCT02610491	The Effect of Hesperidin on Glucose / Insulin Metabolism	53 male and female of 18–65 age were given Hesperidin Citrus peel extract	Completed
NCT02035592	The Health Effects of Blueberry Anthocyanins in Metabolic Syndrome (the CIRCLES-study) (CIRCLES)	144 male and female patients of 50–74 age with metabolic syndrome were given 26 g of freeze-dried blueberry powder per day (6 mouths)	Completed
NCT04731987	Orange Juice, Hesperidin and Their Role in Vascular Health Benefit (HESPER-HEALTH)	42 subjects between 40 and 65 years old with predisposition to cardiovascular disease will consume daily 330 mL of orange juice naturally rich in hesperidin (drink A) during 6 weeks	Recruiting
NCT01617603	A Comparison Chocolate with and Without High Cocoa Solids in Patients with Type 2 Diabetes in a Randomised Clinical Trial	62 male and female patients of 45–75 age with type 2 diabetes were given high polyphenol milk chocolate containing approximately 1 mg/g of epicatechin	Completed
NCT04053569	Grape Polyphenols and Metabolic Syndrome (PolyGrape)	40 obese male and female of 30–65 age were given grape (5 g/kg) administered for four weeks	Active, not recruiting

NCT numbers refer to the source of www.clinicalTrails.gov.

In a randomised, double-blind controlled trial, patients with non-alcoholic fatty liver disease (NAFLD) were given either 250 mg of genistein daily or placebo daily for 8 weeks. Results compared with placebo, showed that the genistein group had lower serum insulin levels and HOMA-IR. In addition, the serum levels of MDA, TNF-α and IL-6 were also lower. In conclusion, oral administration of 250 mg genistein for 8 weeks can reduce IR, oxidation and inflammation, and improve fat metabolism in NAFLD patients. HOMA-IR and fasting insulin levels were significantly reduced in studies using isoflavones or genistein compared to placebo^97^. Cruz et al.^119^ conducted a 2-months study on 45 participants with HOMA-IR > 2.5 and BMI ≥ 30 and ≤ 40 kg/m^2^. It was found that genistein (50 mg/day) improves insulin sensitivity by reshaping intestinal flora, activating skeletal muscle AMPK, and increasing the expression of fatty acid oxidation genes. Microarray analysis of gene expression in skeletal muscle biopsies revealed that genes associated with fatty acid oxidation, including those regulated by AMPK, were up-regulated in the genistein group.

Current treatment of IR mainly recommends lifestyle modification as well as improving insulin sensitivity. However, some antidiabetic drugs can relieve IR directly or indirectly. For example, in another study, glimepiride increased adiponectin levels and decreased IR as measured by HOMA-IR^120^. Metformin has been used as first-line therapy for the treatment of type 2 diabetes for decades. Metformin increases insulin sensitivity and decreases IR through direct and indirect effects on mediators of the initial stages of the insulin signalling pathway, activation of AMPK, mediators of GLUT4 trafficking and translocation, and complex AMPK-dependent and -independent epigenetic modifications^121^. Also, metformin appears to improve whole-body and peripheral IR in youth who are overweight/obese with type 1 diabetes^122^. Several drugs targeting hepatic lipid metabolism and energy metabolism modulators have beneficial effects on IR, such as ketohexokinase inhibitors, PTP1B inhibitors, and fibroblast growth factor analogues. In clinical practice, PPAR-γ agonists such as rosiglitazone and pioglitazone have been used for a long time to manage IR. They decrease the activation of IL-6 and TNF and regulate the synthesis of adiponectin^87^. Thiazolidinedione (another PPAR-γ agonist) has been limited due to severe hepatotoxicity and cardiovascular side effects, although it is approved for clinical practice. Therefore, researchers are now trying to find out natural compounds for the management of abnormalities associated with metabolic syndrome with high efficacy and non or fewer side effects. As it has been established earlier that PPAR-γ ligands have a promising role in the treatment of metabolic syndrome, therefore much more attention has been paid towards newer and safer natural PPAR-γ agonist compounds^123^. More interestingly, flavonoids are able to activate PPAR-γ, modulate NF-kB factors, decrease IR and manage metabolic syndrome^8^^,^^124^. Overall, these results on preclinical and clinical models indicate that the effects of flavonoids in IR are worthy of emphasis and investigation in future clinical studies. Flavonoids deriving from food and plants are used as supplements or nutraceutical products, but the challenging aspect of isolation of flavonoids, their bioavailability and stability, should be resolved via different techniques.

## Conclusion

IR is not only a pre-disease state, but also co-occurs with multiple diseases, such as obesity, diabetes, and metabolic syndrome. At the same time, IR occupies a large part in the pathophysiological state of diabetes. Many research teams are trying to solve IR while conquering diabetes. Indeed, flavonoids have long been recognised to possess important biological activities in human cells. A well-balanced diet, low intake of salt and sugar, intake of micronutrients, unsaturated fats, fruits, vegetables, and moderate physical activity are all essential for maintaining good health and prevention of noncommunicable ailments. Consumption of balanced and a flavonoid-rich diet low in carbohydrates and saturated fats are critical factors to decrease IR and manage metabolic syndrome.

This review has focussed on the mechanism by which flavonoids decrease IR and describes the biological activities of various flavonoids. We found that hydroxyl substitution at the 5 or 7 position of the A ring and at the 3′-5′ position of the B ring in the flavonoid skeleton played a beneficial role in reducing IR of the compounds. This is of great help to pharmaceutical work, and researchers can use it as a reference in design of drugs. Due to their wide range of activities, flavonoids have great potential in clinical application and clinical development, providing a basis for researchers and clinical workers. Although flavonoid compounds have shown powerful effects *in vitro* and animal experiments, such as decreasing IR, reducing blood glucose, and cardiovascular protection, in vivo experiments and clinical trials are lacking, so researchers still need to make further efforts. Few medications are currently being used to treat IR, but there is one drug in clinical phase I (Kareus Therapeutics’ KU-5039 targets AK2, PRKAB1, AK4 and AK1 to treat IR) and one drug in clinical phase II (Xortx Therapeutics’ Oxy- pure). The stage of clinical research by National Centre For complementa on Hesperetin to decrease IR is unknown, but the rich activity of flavonoids provides it with great potential. Although many of these compounds have not yet entered the clinic, the chemical synthesis and optimisation of flavonoids and their further derivatives are necessary in order to decrease IR and treat diabetes and metabolism-related diseases more effectively in the future. In addition, this class of compounds ameliorates the target of IR, providing great value for the design and development of more effective compounds to support human health.
